# Superpersistent currents and whispering gallery modes in relativistic quantum chaotic systems

**DOI:** 10.1038/srep08963

**Published:** 2015-03-11

**Authors:** Hongya Xu, Liang Huang, Ying-Cheng Lai, Celso Grebogi

**Affiliations:** 1 School of Electrical, Computer and Energy Engineering, Arizona State University, Tempe, AZ 85287, USA; 2 School of Physical Science and Technology and Key Laboratory for Magnetism and Magnetic Materials of MOE, Lanzhou University, Lanzhou, Gansu 730000, China; 3 Department of Physics, Arizona State University, Tempe, Arizona 85287, USA; 4 Institute for Complex Systems and Mathematical Biology, King's College, University of Aberdeen, Aberdeen AB24 3UE, UK

## Abstract

Persistent currents (PCs), one of the most intriguing manifestations of the Aharonov-Bohm (AB) effect, are known to vanish for Schrödinger particles in the presence of random scatterings, e.g., due to classical chaos. But would this still be the case for Dirac fermions? Addressing this question is of significant value due to the tremendous recent interest in two-dimensional Dirac materials. We investigate relativistic quantum AB rings threaded by a magnetic flux and find that PCs are extremely robust. Even for highly asymmetric rings that host fully developed classical chaos, the amplitudes of PCs are of the same order of magnitude as those for integrable rings, henceforth the term *superpersistent currents* (SPCs). A striking finding is that the SPCs can be attributed to a robust type of relativistic quantum states, i.e., *Dirac whispering gallery modes* (WGMs) that carry large angular momenta and travel along the boundaries. We propose an experimental scheme using topological insulators to observe and characterize Dirac WGMs and SPCs, and speculate that these features can potentially be the base for a new class of relativistic qubit systems. Our discovery of WGMs in relativistic quantum systems is remarkable because, although WGMs are common in photonic systems, they are relatively rare in electronic systems.

A remarkable phenomenon in the quantum world is persistent currents (PCs), permanent currents without any external source^1^, which are generated by the Aharonov-Bohm (AB) effect^2^ in non-superconducting systems. PCs have been observed experimentally in metallic^3^,^4^,^5^,^6^,^7^ and semiconductor^8^,^9^,^10^ rings in the mesoscopic regime. Theoretical efforts have been focused on the effects of bulk disorders^11^,^12^,^13^, electron-electron interactions^14^,^15^,^16^, spin-orbital interactions^17^,^18^, and electromagnetic radiation^19^,^20^ on PCs, typically studied in the diffusive regime using idealized circular-symmetric rings and cylinders. Rapid advances in nanotechnology have made it feasible to fabricate mesoscopic devices with mean free path larger than their sizes at sufficiently low temperatures (the ballistic transport regime)^21^. The AB system can thus be modeled as a quantum ballistic billiard in which the particles are scattered at the boundaries of the domain. As a result, the boundary shape becomes highly relevant. In experiments, uncontrollable boundary imperfections are inevitable^22^,^23^ even when there are no bulk disorders. It is thus of interest to study the effects of boundaries, e.g., those that generate chaos in the classical limit, on PCs. In general, an asymmetric boundary destroys angular momentum conservation and introduces irregular scattering. Theoretical^24^,^25^,^26^,^27^,^28^,^29^ and experimental^22^ studies have shown that, similar to the effects of bulk disorder, symmetry breaking of the boundary can result in opening of gaps at the degeneracy points of the energy levels, leading to level repulsion, a typical manifestation of classical chaos. Energy gap opening can diminish AB oscillations through pinning of the corresponding states, leading to vanishing PCs. Since fully chaotic domains are associated with a strong degree of symmetry breaking, PCs are not expected to arise^30^,^31^. In nonrelativistic quantum systems governed by the Schrödinger equation, PCs are thus *fragile*.

In this paper, we address the question of whether, in relativistic quantum systems, PCs can arise and sustain in the presence of symmetry-breaking perturbations. Besides the importance of this question to fundamental physics, our work was motivated by the tremendous recent research on two-dimensional Dirac materials^32^ such as graphene^33^,^34^,^35^,^36^,^37^,^38^,^39^, topological insulators^40^, molybdenum disulfide (MoS_2_)^41^,^42^, HITP [Ni_3_(HITP)_2_]^43^, and topological Dirac semimetals^44^,^45^. The physics of these materials is governed by the Dirac equation. This is thus interest in investigating relativistic PCs in Dirac fermion systems^46^,^47^,^48^,^49^,^50^,^51^,^52^,^53^,^54^,^55^,^56^,^57^. Existing theoretical works on relativistic PCs, however, assumed idealized circular-symmetric rings in the ballistic limit. Whether AB oscillations and consequently relativistic PCs can exist in asymmetric rings that exhibit chaos in the classical limit is a fundamental question. Our finding is that, even in the presence of significant boundary deformations so that the classical dynamics becomes fully chaotic, robust PCs can occur in relativistic quantum, Dirac fermion systems, henceforth the term *superpersistent currents* (SPCs). A more striking finding is that SPCs are generated by localized states at the domain boundaries, which are effectively chaotic Dirac whispering gallery modes (WGMs) that carry larger angular momenta. WhileWGMs are common in photonic systems^58^,^59^,^60^,^61^, its emergence in electronic systems^62^, especially in relativistic quantum systems, is rare and surprising. We develop a physical understanding of the counterintuitive phenomenon of SPCs by analytically exploiting the properties of the spinor wavefunctions in an idealized relativistic quantum system.

The significance of our results lies in the perspective of observing SPCs in the presence of strong random scattering, implying that they may occur in systems of size beyond the mesoscopic limit. This can potentially be a relativistic quantum phenomenon occurring at relatively large scales. There can be significant applications of SPCs in quantum information processing, for which we speculate on a scheme of Dirac WGM-based qubit. We note that, previous experimental studies of AB oscillations in graphene^63^ and topological insulators^64^,^65^ make it possible to experimentally test the phenomena of DiracWGMs and SPCs that we predict in this paper. To motivate experimental verification, we propose a feasible scheme using three-dimensional topological insulators.

## Theoretical model of the relativistic AB system

**Figure 1:**
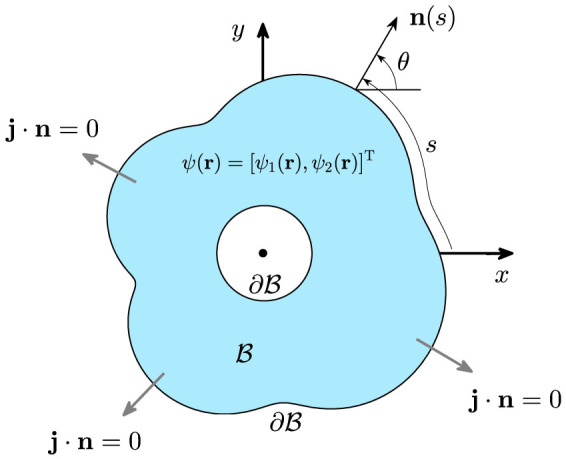
Illustration of a chaotic ring domain with boundary parameterized by the arc length *s*. For motion of massless Dirac fermion inside the domain, the boundary condition is of the zero-flux type, i.e., no outward current at any point *s*: ***j*** · ***n*** = 0.

Consider a single massless spin-half particle of charge −*q* confined by hard walls in a domain 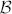 with a ring topology in the plane ***r*** = (*x*, *y*), as shown in Fig. 1. Applying a single line of magnetic flux (AB flux) Φ at its origin and utilizing an infinite mass term outside the domain to model the confinement, we obtain the following Hamiltonian in the position representation:
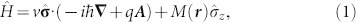
where *v* is the Fermi velocity, 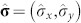 and 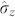 are the Pauli matrices, and *M* = 0 in the ring domain but *M* = ∞ otherwise (for hard-wall confinement - used previously, e.g., in the study of graphene rings^66^, graphene quantum dots^67^, and topological insulator quantum dots^68^). The vector potential ***A***(***r***) can be chosen as any vector field satisfying 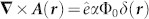, where 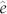 is the unit vector perpendicular to 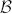 and *α* ≡ Φ/Φ_0_ is the dimensionless quantum flux parameter, with 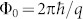 being the flux quantum. The Hamiltonian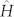 acting on the two-component spinor wave-function *ψ*(***r***) = [*ψ*_1_, *ψ*_2_]^T^ has eigenvalue *E*:
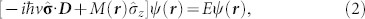
where ***D*** = **∇** + *i****a*** is a compact notation for the covariant derivative with ***a*** ≡ 2*π****A***/Φ_0_.

In Eq. (2), *Mσ_z_* represents the mass potential that takes into account the Klein tunneling effect, which can confine massless Dirac fermions in a finite domain. Such a confinement itself will break the time-reversal symmetry (*T*-breaking) in absence of any external magnetic field. However, the *T*-breaking due to mass potential confinement is different from that due to magnetic field in a classical picture in which no Lorenz force acts on the particle so that the geodesic motions are still characterized by straight lines within the confined domain. In relativistic quantum theory of electrons, magnetic field is introduced through the form of minimal coupling in the corresponding vector potential that is different from the mass term. As a result, in the conventional (3 + 1)-dimensional spacetime, the mass itself cannot break the time-reversal symmetry. However, in (2 + 1)-dimensional systems studied in this paper (as for two-dimensional Dirac materials such as graphene and topological insulators), the mass term will induce chiral anomaly and hence *T*-breaking. Thus the *T*-breaking caused by mass confinement has different features from that due to the magnetic field^69^,^70^.

To better understand the physical origin of the mass confinement term *Mσ_z_*, we consider a single particle in absence of magnetic field and compare the following two situations: (a) the particle is in a Dirac ring with hard-wall confinement of mass potential and (b) the particle is in a Schrödinger ring with the conventional, electrical potential (hard-wall) confinement. We assume that the classical orbits are identical for both cases. Apparently, in case (a), the *T*-breaking is intrinsic but there is no *T*-breaking in case (b). In fact, as indicated by Sir Berry^71^, the semiclassical origin of *T*-breaking induced by mass confinement is quite intriguing. One aim of our work is to uncover new and interesting phenomena caused by the mass confinement *Mσ_z_* in (2 + 1)-dimensional Dirac systems. As we argue and demonstrate later, this type of confinement can be experimentally realized by exploiting the surface states of three-dimensional topological insulators, where the mass term in the Dirac equation is originated from a Zeeman term induced by local exchange coupling.

Some basic properties of Eq. (2) are the following. Firstly, the confinement condition of imposing infinite mass outside 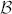 naturally takes into account the Klein paradox for relativistic quantum particles and thus guarantees that our study is conducted in the single-particle framework, which is relevant to the *intrinsic* physics of a single Dirac cone in graphene or topological insulator. Secondly, both reduced spatial dimension together with mass confinement and applied magnetic flux can break the time-reversal symmetry of 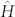: 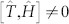 if *M* ≠ 0 or *α* ≠ 0, where 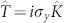 and 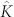 denotes complex conjugate. Thirdly, for *M* = 0 and ***A*** = 0 in Eq. (2) (i.e. free massless particle), there exist plane wave solutions whose positive energy part has the following form:
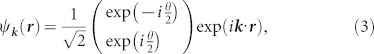
where ***k*** is a wave vector that makes an angle *θ* with the *x* axis. Fourthly, by using the Dirac equation 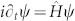 and defining *ρ* = *ψ*^†^*ψ* as the local probability density, we have the following continuity equation 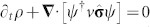. It is therefore natural to define 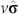 as the local current operator, so the local current density in state *ψ*(***r***) is given by 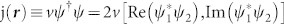.

To obtain solutions of Eq. (2), a proper treatment of the boundary condition is necessary. As done in previous works, we use the infinite mass (also called Berry-Mondragon^71^ or chiralbag^72^,^73^,^74^) boundary condition
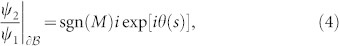
where sgn(·) stands for the signum function and *θ*(*s*) denotes the angle made by the outward unit normal ***n*** with the *x* axis at an arbitrary boundary point *s*, as shown in Fig. 1. Substituting Eq. (4) into the current density formula, one can show that the boundary current ***j***(*s*) = 2*v*|*ψ*_1_|^2^(− sin *θ*, cos *θ*) is polarized along the boundary: *clockwise* and *counterclockwise* for the inner and outer edges, respectively. It is remarkable that this polarized property is independent of the shape of the confinement potential *M*(***r***) and is thus *topologically protected* from irregular boundary scatterings, even though the magnitude of the edge current can be affected.

An analysis of the general properties of the *α* (magnetic flux) dependent relativistic quantum spectrum {*E_j_*(*α*)}, as determined by Eq. (2) under the boundary condition Eq. (4), reveals that the first “Brillouin zone” is given by −1/2 ≤ *α* ≤ 1/2 (Supplementary Note 1). To calculate a large number of relativistic eigenvalues and eigenstates with high accuracy, we use the conformal-mapping method^75^,^76^,^77^ (Supplementary Note 2).

## Whispering gallery modes and superpersistent currents

**Figure 2:**
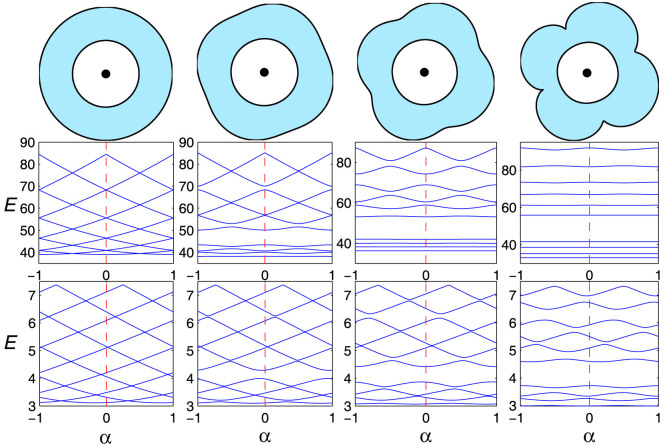
Top panels: domain shape with classical dynamics ranging from integrable (*a* = 0; left most panel) and mixed (*a* = 0.25 and 0.5; middle two panels) to chaotic (*a* = 1.0; right most panel). Middle panels: nonrelativistic AB oscillations (energy-flux dispersions). Bottom panels: relativistic AB oscillations.

To demonstrate our findings, we deform a circular ring domain *ξ* = 0.5 ≤ *r* ≤ 1, using the mapping *w*(*z*) = *h*[*z* + 0.05*az*^2^ + 0.18*a* exp(*iω*)*z*^5^], where *ω* = *π*/2, *a* ∈ [0, 1] is the deformation parameter that controls the classical dynamics. Specifically, when increasing *a* from zero to unity the deformed ring will undergo a transition from being regular to mixed and finally to being fully chaotic. The normalization coefficient 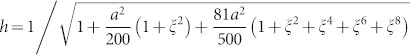 guarantees that the domain area is invariant for arbitrary values of the deformation parameters {*a*, *ω*, *ξ*}. Four representative domains are shown in the top row in Fig. 2 where, classically, the left most domain is integrable, the right most domain is fully chaotic, and the two middle domains have mixed phase space. The middle and bottom rows of Fig. 2 show the lowest 10 energy levels as functions of the quantum flux parameter *α*, i.e., energy-flux dispersions, for Schrödinger and Dirac particles, respectively. We see that *E_j_*(*α*) = *E_j_*(−*α*) holds for the Schrödinger particle, but for the Dirac fermion, the symmetry is broken: *E_j_*(*α*) ≠ *E*(−*α*). However, for both nonrelativistic and relativistic spectra, we have *E_j_*(*α*) = *E_j_*(*α* + 1). For the circular-symmetric ring (*a* = 0), AB oscillations in the energy levels have the period Φ_0_ (i.e., *α* = 1) and there are level crossings. Making the domain less symmetric by tuning up the value of the deformation parameter *a* leads to classical mixed phase space (regular and chaotic), and eventually to full chaos (*a* = 1). We see that, for the Schrödinger particle, emergence of a chaotic component in the classical space leads to opening of energy gaps, generating level repulsion and flattening the AB oscillations associated with the corresponding energy levels. Surprisingly, for the Dirac fermion, the AB oscillations are much more robust against asymmetric deformations. In particular, for the fully chaotic case, AB oscillations for the Schrödinger particle disappear almost entirely while those for the Dirac fermion persist with amplitudes of the same order of magnitudes as the integrable case.

**Figure 3:**
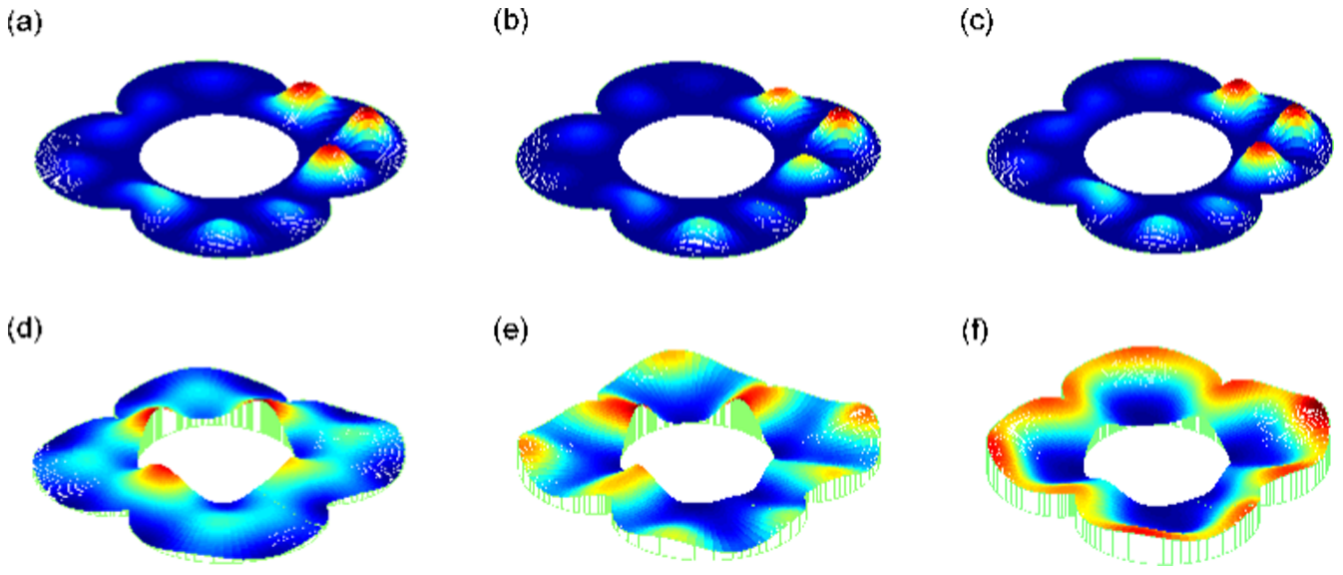
Probability distribution of the 10th eigenstate for (a–c) nonrelativistic and (d–f) relativistic AB chaotic billiard for *α* = −1/4, 0, 1/4, respectively.

We now present evidence of Dirac WGMs for the case of fully chaotic AB ring domain. By examining the eigenstates, we note that, for low energy levels, the Schrödinger particle is strongly localized throughout the domain, as shown in Figs. 3(a–c), leading to a flat energy-flux dispersion. However, the Dirac fermion typically travels around the ring's boundaries, forming relativistic WGMs that persist under irregular boundary scattering due to chaos and are magnetic-flux dependent, as shown in Fig. 3(d–f). Conventional wisdom for Schrödinger particle stipulates that asymmetry in the domain geometry can mix/couple well-defined angular momentum states, opening energy gaps and leading to localization of lower states in the entire domain region, so AB oscillations would vanish, as demonstrated both theoretically and experimentally^22^,^24^. However, for Dirac fermion, this picture breaks down - there are robust AB oscillations even in the fully chaotic domain and the particle tends to execute motions corresponding to WGMs.

**Figure 4:**
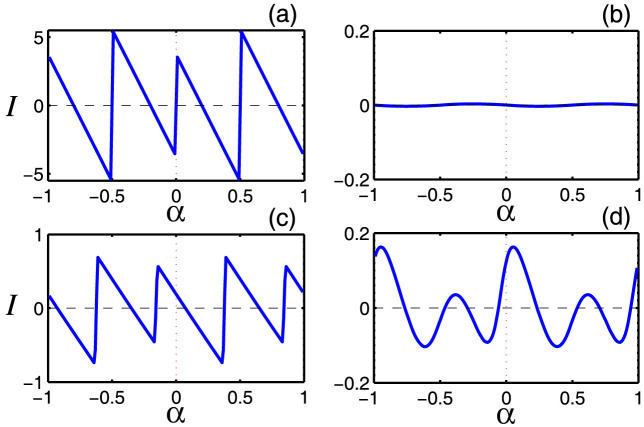
Persistent current (PC) as a function of the quantum flux parameter *α* from five lowest states (including spin) for nonrelativistic (a,b) and relativistic (c,d) cases. The domain is integrable for (a,c) and chaotic for (b,d).

The robust AB oscillations in chaotic Dirac rings lead to SPCs. The total persistent current can be calculated at zero temperature through^1^,^66^ 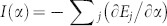, where the sum runs over all occupied states with *E_j_* > 0. Due to periodicity in the energy: *E_j_*(*α*) = *E_j_*(*α* + 1), the current is also periodic in *α* with the fundamental period *α* = 1. Figure 4 shows, for the nonrelativistic (top panels) and relativistic (bottom panels) cases, PCs resulted from the lowest three states (including spin) in regular (left column) and chaotic (right column) rings. We see that, for classical integrable dynamics, PC oscillations display a common sawtooth form. However, at zero flux, PC is zero for the nonrelativistic case [Fig. 4 (a)], while it has a finite value for the relativistic case due to breaking of the time-reversal symmetry. In the chaotic case, the oscillations become smooth due to level repulsion in the corresponding energy-flux dispersion pattern. As a result, PCs carried by the Schrödinger particle practically vanish as compared with the integrable case but, strikingly, the Dirac fermion still carries a persistent current with amplitude of the same order of magnitude as that for the integrable case - SPCs. Intuitively, SPCs carried by the Dirac fermion as an “exceptional” magnetic response are associated with the chaotic Dirac WGMs exemplified in Fig. 3.

## Origin of WGMs and SPCs

**Figure 5:**
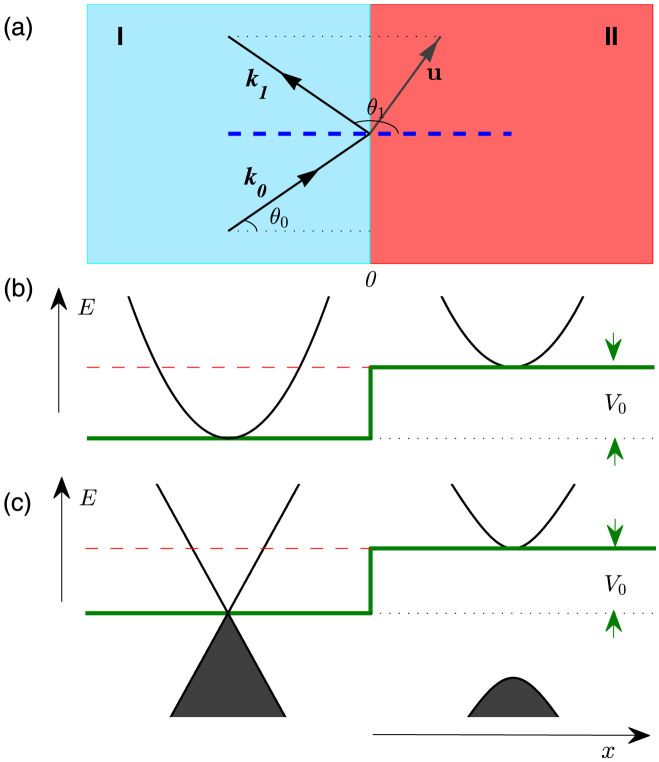
Incident, reflected and transmitted local plane waves at a potential jump.

The origin of the “exceptional” magnetic response of the chaotic Dirac fermion can be understood through the behavior of the current carried by the particle at the boundary interface. We have developed an analytic understanding to predict the occurrence of Dirac WGMs and, consequently, SPCs. In particular, we consider the following problem: a plane wave incident obliquely on a straight potential jump *M*(*x*, *y*) given by
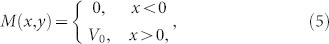
as shown in Fig. 5. Without loss of generality, we let the incident wave *ψ^i^* be described by the wave vector ***k***_0_ = (*k* cos *θ*_0_, *k* sin *θ*_0_), the reflected wave *ψ^r^* by ***k***_1_ = (*k* cos *θ*_1_, *k* sin *θ*_1_, and the transmitted wave *ψ^t^* by ***u*** = (*iq*, *K*), where *θ*_1_ = *π* − *θ*_0_ and *K* ≡ *k* sin *θ*_0_. We focus on the situation where the energy of the incident wave satisfies *E* < *V*_0_, which corresponds to the total reflection case.

Referring to Fig. 5, we have that the wave in region I (*x* < 0) can be written as
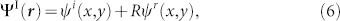
and the wave in region II is
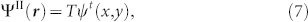
where the coefficients *R* and *T* are to be determined by matching the waves at *x* = 0. In the following, we treat the Schrödinger scalar wave and Dirac spinor wave separately.

## Schrödinger scalar plane wave

For the nonrelativistic quantum case as shown in Fig. 5(b), we have, in region I (*x* < 0),
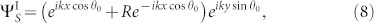
and in region II (*x* > 0),
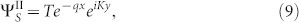
where *q* and *K* are related to each other through
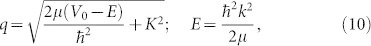
with *µ* denoting the mass of the particle. Matching the waves and their derivatives at *x* = 0, we obtain
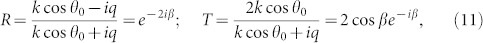
where the parameter *β* is defined through
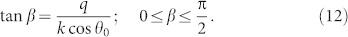
Given the wave function Ψ*_S_*(***r***), the associated probability current density is
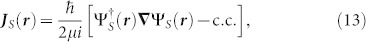
where c.c. denotes complex conjugate. We therefore obtain, in region I,
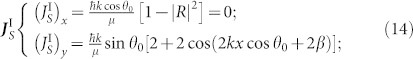
and in region II,
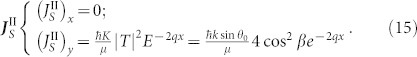
In region I, the *y* component of the probability current is the sum of two terms: (1) the term resulting from the sum of the currents associated with the incident and reflected waves, and (2) the term containing the factor cos(2*kx* cos *θ*_0_ + 2*β*) that accounts for the interference between the incident and reflected waves. In region II, the probability current is also parallel to the *y*-axis, and it decays exponentially as an evanescent wave. Note that 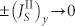 as *q* → ∞ (i.e., *V*_0_ → ∞).

## Planar Dirac spinor wave

The relativistic case is shown in Fig. 5(c). We proceed in the same manner as for the nonrelativistic case. Expressing the wave in terms of massless spinor planar waves that are solutions of the Dirac equation, we have, in region I (*x* < 0),
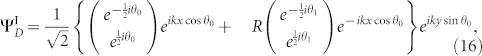
and in region II (*x* > 0),
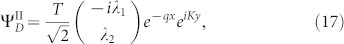
where
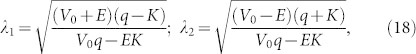
and
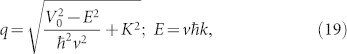
with *v* being the Fermi velocity.

**Figure 6:**
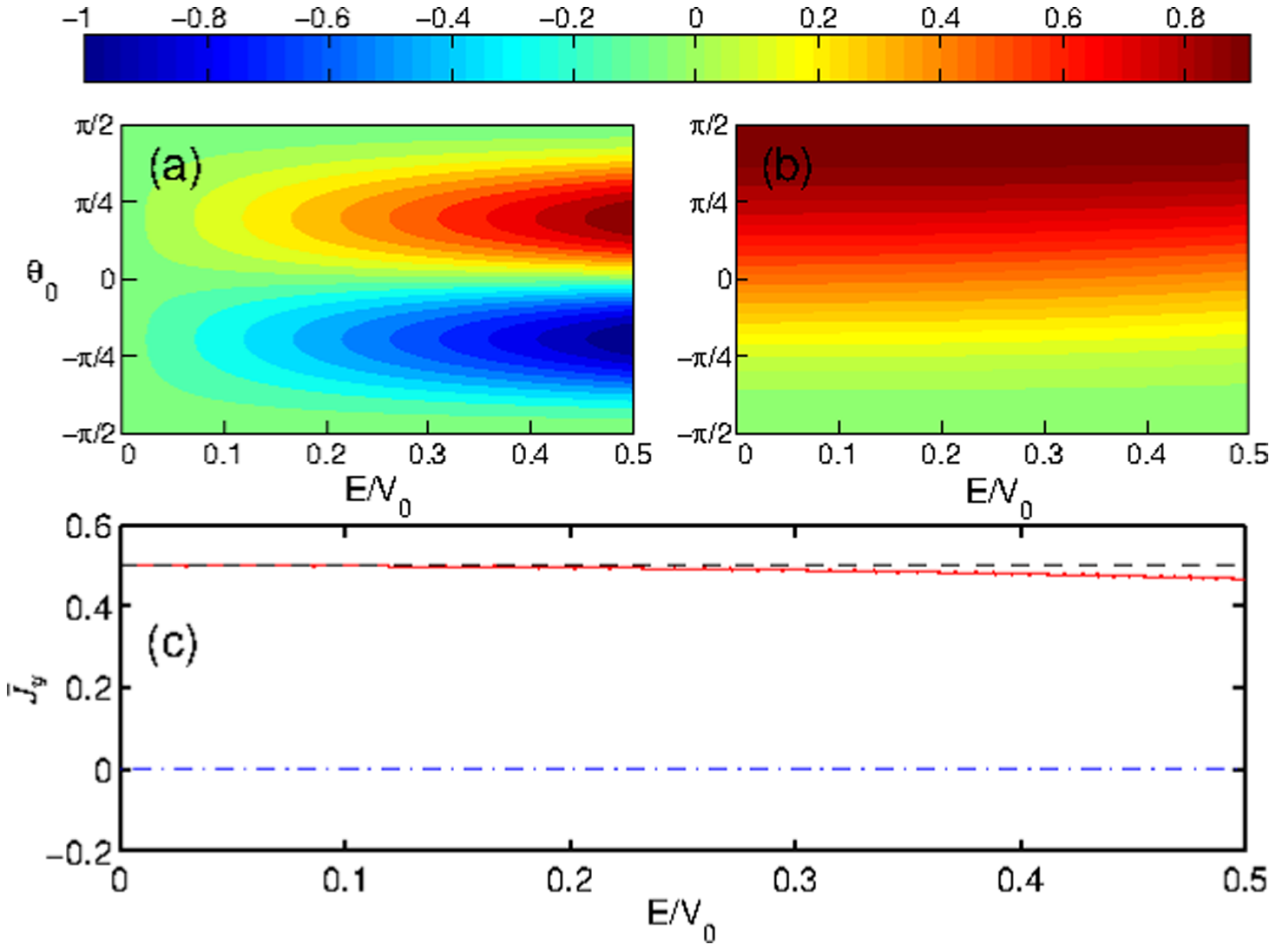
Comparison of the *y*-component of the current density (coded by colors) as a function of the incident angle *θ*_0_ and the height of the reduced potential barrier *E*/*V*_0_ between the nonrelativistic (a) and relativistic (b) cases at the interface (*x* = 0). (c) The *y*-component of the current density averaged over all possible incident angles *θ*_0_ as a function of *E*/*V*_0_: blue dash-dotted (red) curve is for the nonrelativistic (relativistic) case and the dash black line denotes the theoretical estimation of the relativistic case based on Eq. (26) with the assumption 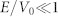. In both cases, the values of the current density have been normalized by the respective maxima.

Matching boundary conditions at *x* = 0, we obtain
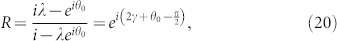
where the parameters *γ* and *λ* are defined through
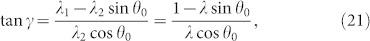

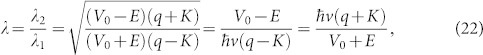


and
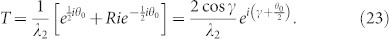
For the spinor wave Ψ*_D_*(***r***) describing a Dirac fermion in two dimensions, the corresponding probability current density is
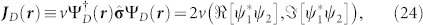
where 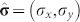 and Ψ*_D_* = [*ψ*_1_, *ψ*_2_]^T^. We have, in region I,
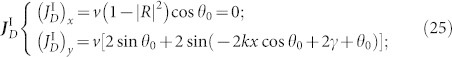
and in region II,
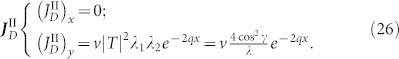
From Eqs. (14) – (15) and Eqs. (25) – (26), we observe identical behaviors in the normal current densities (i.e., the *x*-component) for both nonrelativistic and relativistic cases, but there is a significant difference in the transverse current densities (i.e., the *y*-component). In particular, as *E*/*V*_0_ → 0 (the hard-wall limit), we have 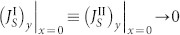 for the nonrelativistic case, while 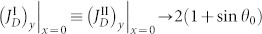 for the relativistic case. In addition, it is apparent from Eqs. (14) – (15) that (*J_S_*)*_y_* is antisymmetric (odd function) with respect to *θ*_0_ so that its average over all possible incident angles, 
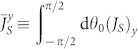
, is zero. The averaged transverse current density 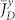 in the relativistic case, however, tends to a finite value in the hard-wall limit. A schematic comparison of the *y* component of the probability current density ***J*** at the interface (*x* = 0) as a function of the incident angle *θ*_0_ and the magnitude of the reduced incident energy *E*/*V*_0_ between the Schrödinger and Dirac cases is shown in Fig. 6. We see that the nonrelativistic transverse current density (*J_S_*)*_y_* is antisymmetric with respect to *θ*_0_, leading to zero contribution to 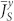, while the relativistic transverse current density (*J_D_*)*_y_* is a nonnegative monotonic function of *θ*_0_ so that there exists a finite transverse current even for a fully chaotic ring when all possible incident angles are taken into account. Such a finite averaged transverse current density 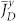 with magnitude one half the maximum makes it possible to form chaotic Dirac whispering gallery modes that carry considerable directional currents. This is the fundamental mechanism for the phenomenon of SPCs.

Note that, although Eqs. (25) and (26) appear different in form, at the interfact (*x* = 0) they give exactly the same current density (see Supplementary Note 3). For *V*_0_ → ∞, the hard-wall boundary condition is restored (the infinity-mass boundary condition). Physically the counterintuitive phenomenon can be understood, as follows. The incoming wave from the metal region (mass term *M* = 0) is spin polarized along its momentum (current) direction. After entering the insulator region, a finite mass term acting on *σ_z_* will change the direction of the spin and hence affect the current via the spin-momentum locking term **k** · *σ*. The relevant Hall-like phenomenon associated with the *T*-breaking mass potential has been uncovered in a very recent work^78^. In addition, we note that the results do not depend on the special form of the wave function in region II (although such a symmetric form is convenient for analysis). In fact, we obtain the same results^38^ when choosing the wavefunction to have the form ~ [1, *C*(*E*)]*^T^*.

## Experimental scheme

A possible experimental scheme to observe and characterize Dirac WGMs and SPCs is, as follows. A 3D topological insulator supports a (2D) gapless state on its surface, with low-energy excitations described by the massless Dirac Hamiltonian^40^,^79^ 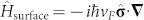, where 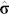 characterizes the spin. The surface electronic structure is similar to that of graphene, except that there is only a single Dirac point. Different from graphene, the Dirac surface states of a topological insulator are associated with strong spin-orbit interactions. In spintronics applications of topological insulators, it is desirable to introduce a gap into the surface states. This can be done by breaking the time-reversal symmetry using a ferromagnet insulator (FMI) deposited on the top of a topological insulator^68^. The exchange coupling induced due to proximity to the ferromagnet insulator will give rise to a local exchange field that lifts the Kramers degeneracy at the surface Dirac point and introduces a mass term into the Dirac Hamiltonian. Thus, generally, we have 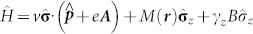, where the vector potential ***A*** accounts for the effect of the external magnetic field 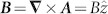, with an additional Zeeman splitting correction in the last term. The controllable mass term 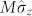, responsible for the local exchange coupling with a FMI cap layer, makes 3D topological insulators a potential experiment platform for observing and characterizing Dirac WGMs and SPCs.

## Robust relativistic qubit based on WGMs

We speculate on a potential application of DiracWGMs in quantum information technology. Similar to the proposal of qubit in a two-dimensional topological insulator based on the two different sets of helical edge states localized at the boundaries^56^ and the idea of charge qubit in a double quantum dot^80^, we present our qubit based on the chaotic Dirac WGMs guided by the inner and outer surfaces in opposite directions. Such an edge degree of freedom can be used to form a two-state system, denoted by the states |on〉 and |off〉 for the *outer* and *inner* chaotic DiracWGMs, respectively. The set {|on〉,|off〉} thus constitutes a complete *diabatic basis*. Generally, these two states correspond to two energy levels {*E_L_*(*α*), *E_U_*(*α*)} with a rather larger difference (mismatch) and can be coupled and superpositioned for different values of the magnetic flux. As a result, two different levels of the system arise, say {*E_L_*(*α*′), *E_U_*(*α*′)}. The corresponding instantaneous eigenstates {|*L*〉,|*U*〉} constitute an *adiabatic basis*. An effective *α*-dependent Hamiltonian describing the flux-tunable qubit can then be written in the adiabatic basis as 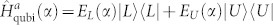, providing a base for exploiting the Dirac WGMs as a qubit system.

## Conclusions

We formulate a relativistic version of AB chaotic billiards to study PCs in Dirac rings. We find that, in contrast to the nonrelativistic quantum counterpart where PCs vanish for chaotic rings, the currents continue to exist in the relativistic chaotic AB rings and, in this sense, they are superpersistent. We demonstrate that SPCs are a consequence of Dirac WGMs, and we develop an analytic understanding of their emergence in relativistic quantumsystems. We also propose that, experimentally, chaotic rings patterned by magnetic domain heterostructures deposited on the surface of a 3D topological insulator can be a feasible scheme to observe and characterize chaotic Dirac WGMs and SPCs. The coexistence of inner and outer chaotic Dirac WGMs naturally forms a flux-tunable two-level system. To investigate the magnetic response of chaotic Dirac fermions is not only fundamental to the emerging field of relativistic quantum chaos, but also relevant to device applications based on Dirac materials.

## Author Contributions

H.-Y.X., Y.-C.L., L.H. and C.G. designed research. H.-Y.X. performed research. H.-Y.X., Y.-C.L. and L.H. analyzed data. H.-Y.X., Y.-C.L., L.H. and C.G. wrote the paper.

## Supplementary Material

Supplementary Information Supplementary Information
