# Supplementary material for: Superpersistent currents and whispering gallery modes in relativistic quantum chaotic systems
Source: Sci Rep. 2015 Mar 11;5:8963. doi: 10.1038/srep08963 (PMC4355680; doi:10.1038/srep08963)
Supplement: Supplementary Information [file srep08963-s1.pdf]

Supplementary Materials for

Superpersistent currents and whispering gallery modes in relativistic quantum chaotic systems

Hongya Xu, Liang Huang, Ying-Cheng Lai, and Celso Grebogi

SUPPLEMENTARY NOTE 1: MAGNETIC FLUX DEPENDENT RELATIVISTIC QUANTUM ENERGY SPECTRA

Before solving the Dirac equation with vector potential, we investigate some general properties of the magnetic flux (characterized by parameter α) dependent relativistic quantum spectrum $\{E_j(\alpha)\}$ as determined by Eq. (1) in the main text, under the boundary condition [Eq. (3) in the main text]. Since the vector potential $\boldsymbol{A}$ depends on the gauge while the Dirac equation is gauge-invariant, we can choose a proper gauge transformation to eliminate explicitly $\boldsymbol{A}$ from the Dirac Hamiltonian $\hat{H} = v\hat{\boldsymbol{\sigma}}\cdot(-i\hbar\boldsymbol{\nabla} + q\boldsymbol{A}) + V(\boldsymbol{r})\hat{\sigma}_z$. An example of such transformation is

$$\phi(\boldsymbol{r}) \equiv \begin{pmatrix} \phi_1 \\ \phi_2 \end{pmatrix} = \psi(\boldsymbol{r}) \times \exp\left[-i\frac{2\pi}{\Phi_0}\int_{\boldsymbol{r}_0}^{\boldsymbol{r}} \boldsymbol{A}(\boldsymbol{r'})\cdot \mathrm{d}\boldsymbol{r'}\right]. \tag{1}$$

Substituting Eq. (1) into the Dirac equation and taking into account the boundary conditions [Eq. (3) in main text], we obtain

$$\hat{H}_0\phi(\boldsymbol{r}) \equiv \left[-i\hbar v\hat{\boldsymbol{\sigma}}\cdot\boldsymbol{\nabla} + V(\boldsymbol{r})\hat{\sigma}_z\right]\phi(\boldsymbol{r}) = E\phi(\boldsymbol{r}), \tag{2}$$

with the following complex boundary condition:

$$\frac{\phi_2}{\phi_1}\Big|_{\partial\mathcal{B}} = \mathrm{sgn}(V) i\exp[i\theta(s)], \tag{3}$$

which is equivalent to the original boundary condition [Eq. (3) in main text]. The nontrivial point here is that, because ψ is single valued, ϕ must be multivalued. As a result, for any circuit C about the flux line, ϕ will acquire a phase factor:

$$\hat{c}\phi = \exp\left[-i\frac{2\pi}{\Phi_0}\oint_C \boldsymbol{A}\cdot\mathrm{d}\boldsymbol{r}\right]\phi = \exp\left(-2\pi i W_C\alpha\right)\phi, \tag{4}$$

where the operator $\hat{c}$ denotes the circuit operation acting on ϕ and W_C $(= \pm1, \pm2, \cdots)$ is the winding number of C. From Eqs. (2)-(4), we can conclude that, because the complex boundary condition does not depend on α, it is not possible to generate the so-called "false T-breaking" only by tuning the quantum flux parameter α, in contrast to the nonrelativistic case [1]. If boundary $\mathcal{B}$ is chosen to be geometrically asymmetric so that there is chaotic dynamics in the classical limit, contrary to nonrelativistic quantum systems, the relativistic quantum spectral statistics are characterized by those of the Gaussian unitary ensemble (GUE), regardless of the value of α. Furthermore, the spectrum $\{E_j(\alpha)\}$ does not possess symmetry with respect to flux reversal ($\alpha \to -\alpha$), in contrast to nonrelativistic quantum spectra as determined by the Schrödinger equation where the symmetry is preserved. This can be shown explicitly, as follows.

For our Dirac ring system, the flux-reversal operator $\hat{f}$ can be represented as

$$\hat{f} = \hat{U}\hat{K}, \tag{5}$$

where $\hat{U}$ is unitary and commutes with $\hat{c}$:

$$[\hat{f}, \hat{c}] = 0. \tag{6}$$

We see that ϕ transforms to

$$\phi' \equiv [\phi'_1, \phi'_2]^{\mathrm{T}} = \hat{f}\phi = \hat{U}\phi^*,$$

and

$$\hat{c}\phi' = \exp[-2\pi i W_C(-\alpha)]\phi'. \tag{7}$$

Thus, in order that $E_j(\alpha) = E_j(-\alpha)$, $\hat{f}$ must satisfy

$$[\hat{f}, \hat{H}_0] = 0, \tag{8}$$

and ϕ' must follow the same boundary condition as Eq. (3). It was pointed out by Berry and Mondragon [1] that, if $V(\boldsymbol{r})$ possesses no geometric symmetry, no operator would exist which satisfies Eq. (5) and Eq. (8) simultaneously, but it may be possible to find an operator with the form given by Eq. (5) that commutes with $\hat{H}_0$ if $V(\boldsymbol{r})$ does possess any geometric symmetry. In our system, however, any such possible operator would not satisfy the pre-condition Eq. (6) at the same time. Consequently, the energy spectrum $\{E_j(\alpha)\}$ does not possess the flux-reversal symmetry, i.e., $E_j(\alpha) \neq E_j(-\alpha)$.

By considering the specific situation of $\hat{U} = i\hat{\sigma}_y$, we see that the flux-reversal symmetry emerges if the sign of the potential V is changed simultaneously, i.e., $\{\alpha \to -\alpha, V \to -V\}$. That is the primary reason that the energy spectrum in graphene is symmetric with respect to $\alpha = 0$ when both valleys are included [2]. From Eqs. (2)-(4), we have

$$E_j(\alpha) = E_j(\alpha + 1). \tag{9}$$

Studying the relativistic AB quantum billiard spectrum in the range $-1/2 \leq \alpha \leq 1/2$ thus suffices, which corresponds to the first "Brillouin zone."

SUPPLEMENTARY NOTE 2: MAGNETIC FLUX DEPENDENT RELATIVISTIC QUANTUM ENERGY SPECTRUM

A. Conformal-mapping method for calculating relativistic quantum eigenvalues and eigenstates in chaotic domains

The idea of employing conformal mapping to calculate quantum eigenenergies and eigenstates was proposed by Robnik and Berry [3, 4] for non-relativistic quantum billiard systems. In a recent work [5], we adopted the method to relativistic quantum billiards governed by the Dirac equation. Here, we further extend the method to relativistic quantum chaotic billiards subject to AB magnetic flux as given by Eq. (2). The basic idea is that, while the Dirac equation together with the boundary condition is generally not separable in the Cartesian coordinates, for circularly

symmetric ring domains analytic solutions can be written down. Given a closed domain with an analytic boundary, we can identify a proper conformal mapping that transforms the domain into a circular ring. The analytic solutions of the Dirac equation in the circular ring can then be transformed back to the original domain to yield a large number of eigenvalues and eigenstates with high accuracy.

We begin by choosing the gauge

$$\boldsymbol{A}(\boldsymbol{r}) = \frac{\alpha}{2\pi}\Phi_0\left(\frac{\partial F}{\partial y}, -\frac{\partial F}{\partial x}, 0\right); \quad F = -\ln|\boldsymbol{r}| \tag{10}$$

and writing the Dirac equation [Eq. (1) in the main text] in the polar coordinates $\boldsymbol{r} = (r, \theta)$ for the closed circular ring domain defined by $V(\boldsymbol{r}) = 0$:

$$\begin{pmatrix} 0 & \exp(-i\phi)\left(\frac{\partial}{\partial r} - \frac{i}{r}\frac{\partial}{\partial \phi} + \frac{\alpha}{r}\right) \\ \exp(i\phi)\left(\frac{\partial}{\partial r} + \frac{i}{r}\frac{\partial}{\partial \phi} - \frac{\alpha}{r}\right) & 0 \end{pmatrix} \times \psi(\boldsymbol{r}) = i\mu\psi(\boldsymbol{r}), \tag{11}$$

where $\mu \equiv E/\hbar v$. For a circularly symmetric ring boundary, we have $[\hat{J}_z, \hat{H}] = 0$, where

$$\hat{J}_z = -i\hbar\partial_\phi + \hbar/2\hat{\sigma}_z$$

is the total angular momentum operator. The solutions of Eq. (11) can thus be written as

$$\psi(\boldsymbol{r}) = \exp[i(l - 1/2)\theta]\begin{pmatrix} \phi(r) \\ i\chi(r)\exp(i\theta) \end{pmatrix}, \tag{12}$$

where $l = \pm 1/2, \pm 3/2, \cdots$ denotes the eigenvalues of $\hat{J}_z$. Substituting Eq. (12) into Eq. (11) yields the following radial equation:

$$\hat{H}_r\begin{pmatrix} \phi(r) \\ \chi(r) \end{pmatrix} = \mu\begin{pmatrix} \phi(r) \\ \chi(r) \end{pmatrix}, \tag{13}$$

with the radial 'Hamiltonian' given by

$$\hat{H}_r = i\hat{\sigma}_y\left(\frac{\mathrm{d}}{\mathrm{d}r} + \frac{1/2}{r}\right) + \hat{\sigma}_x\frac{l+\alpha}{r} = \begin{pmatrix} 0 & \frac{\mathrm{d}}{\mathrm{d}r} + \frac{1/2}{r} + \frac{l+\alpha}{r} \\ -\frac{\mathrm{d}}{\mathrm{d}r} - \frac{1/2}{r} + \frac{l+\alpha}{r} & 0 \end{pmatrix}. \tag{14}$$

Under the similarity transformation: $\hat{\mathcal{P}} = (\hat{\sigma}_x + \hat{\sigma}_z)/\sqrt{2}$, the radial Hamiltonian $\hat{H}_r$ is transformed to

$$\hat{H}'_r = \hat{\mathcal{P}}\hat{H}_r\hat{\mathcal{P}}^\dagger = \begin{pmatrix} -\frac{l+\alpha}{r} & \frac{\mathrm{d}}{\mathrm{d}r} + \frac{1/2}{r} \\ -\frac{\mathrm{d}}{\mathrm{d}r} - \frac{1/2}{r} & \frac{l+\alpha}{r} \end{pmatrix} = i\hat{\sigma}_y\left(\frac{\mathrm{d}}{\mathrm{d}r} + \frac{1/2}{r}\right) + \hat{\sigma}_z\frac{-(l+\alpha)}{r}, \tag{15}$$

with an equivalent mass term $-(l+\alpha)/r$ that is both position and state dependent. Although either the AB flux (α) or the mass (V) can lead to T-breaking, quantitatively their roles in the solutions of the Dirac equation are generally different. As a result, in a Dirac fermion system the effects of fixed T-breaking due to the mass confinement V cannot be completely compensated by those induced by the tunable AB flux α.

From Eqs. (13) and (14), we see that $\phi(r)$ is a solution of the Bessel's equation of order $\bar{l}-1/2$, i.e.,

$$\frac{\mathrm{d}^2\phi}{\mathrm{d}R^2} + \frac{1}{R}\frac{\mathrm{d}\phi}{\mathrm{d}R} + \left[1 - \frac{(\bar{l}-1/2)^2}{R^2}\right]\phi = 0, \tag{16}$$

where $\bar{l} \equiv l + \alpha$ and $R = \mu r$ is the dimensionless radial coordinate. For the ring geometry, $\phi(r)$ can be written as

$$\phi(r) = N\left(H^{(1)}_{\bar{l}-1/2}(\mu r) + \beta H^{(2)}_{\bar{l}-1/2}(\mu r)\right), \tag{17}$$

where $H^{(1,2)}_{\nu}$ denote the ν-th order Hankel functions of the (first, second) kind, N and β are undetermined coefficients. Substituting Eq. (17) back into Eq. (13) and using the relations

$$H^{(1,2)\prime}_{\nu}(x) - \nu H^{(1,2)}_{\nu}(x)/x = -H^{(1,2)}_{\nu+1}(x),$$

we obtain the second radial component

$$\chi(r) = N\left(H^{(1)}_{\bar{l}+1/2}(\mu r) + \beta H^{(2)}_{\bar{l}+1/2}(\mu r)\right). \tag{18}$$

Without loss of generality, we assume that the outer radius of the ring is unity and the inner radius is $\xi \in (0,1)$. Imposing the boundary condition [Eq. (3) in main text] on the wavefunction $\psi(\boldsymbol{r})$ with the expressions of Eqs. (12), (17), and (18), and taking into account their orthonormal property, we get

$$\beta = -\frac{\mathcal{H}^{(1)}_{+}(\mu_j\xi)}{\mathcal{H}^{(2)}_{+}(\mu_j\xi)} = -\frac{\mathcal{H}^{(1)}_{-}(\mu_j)}{\mathcal{H}^{(2)}_{-}(\mu_j)}, \tag{19}$$

with the compact notations $j \equiv (\bar{l}, n)$ and

$$\mathcal{H}^{(m)}_{\pm}(x) \equiv H^{(m)}_{\bar{l}+1/2}(x) \pm H^{(m)}_{\bar{l}-1/2}(x); \quad \text{for } m = 1, 2,$$

as well as the normalization constants

$$|N|^{-2} = 2\pi\int_{\xi}^{1}\mathrm{d}r r\left[\left|H^{(1)}_{\bar{l}-1/2}(\mu_j r) + \beta H^{(2)}_{\bar{l}-1/2}(\mu_j r)\right|^2 + \left|H^{(1)}_{\bar{l}+1/2}(\mu_j r) + \beta H^{(2)}_{\bar{l}+1/2}(\mu_j r)\right|^2\right]. \tag{20}$$

The quantity μ_j in Eqs. (19) and (20) satisfies the (compact) energy eigenvalue equation

$$\begin{vmatrix} \mathcal{J}_{-}(\mu) & \mathcal{Y}_{-}(\mu) \\ \mathcal{J}_{+}(\mu\xi) & \mathcal{Y}_{+}(\mu\xi) \end{vmatrix} = 0, \tag{21}$$

$$\begin{aligned} \mathcal{J}_{\pm}(x) &\equiv J_{\bar{l}+1/2}(x) \pm J_{\bar{l}-1/2}(x), \\ \mathcal{Y}_{\pm} &\equiv Y_{\bar{l}+1/2}(x) \pm Y_{\bar{l}-1/2}(x), \end{aligned}$$

with J and Y being the Bessel functions of the first and second kind, respectively.

We have so far obtained the complete (positive-energy part) solutions $\{\psi_j, \mu_j\}$ to Eq. (11) in the circular ring domain under the boundary condition. The eigenfunctions $\psi_j \equiv \langle\boldsymbol{r}|j\rangle$ satisfy

$$\langle i|j\rangle \equiv \iint_{\mathcal{B}} \mathrm{d}\boldsymbol{r}^2\langle i|\boldsymbol{r}\rangle\langle\boldsymbol{r}|j\rangle = \iint_{\mathcal{B}} \mathrm{d}\boldsymbol{r}^2\psi_i^{\dagger}(\boldsymbol{r})\psi_j(\boldsymbol{r}) = \delta_{ij},$$

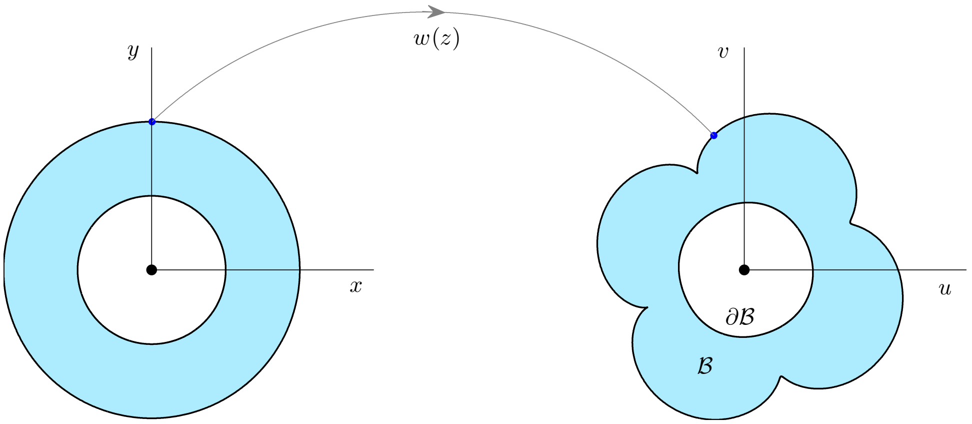

FIG. 1. Conformal transformation from the unit disc in $z = x + iy$ (z-plane) to the billiard domain in $w = u + iv$ (w-plane). The boundary is generated by the mapping function Eq. (30) with parameters $a = 1, \omega = \pi/2$, and $\xi = 0.5$. (Adapted from Ref. [4]).

and thus form an orthonormal complete basis for the operator $-i\hat{\boldsymbol{\sigma}} \cdot \boldsymbol{D}$ and its positive integral power $[-i\hat{\boldsymbol{\sigma}} \cdot \boldsymbol{D}]^n$ defined in the same domain under the same boundary condition.

The billiard domain $\mathcal{B}$ in which the Dirac equation is to be solved is defined as a conformal transformation of the circular ring domain in the $w-$plane, as shown in Fig. 1, i.e.,

$$u(x, y) + iv(x, y) = w(z) \equiv w(re^{i\phi}), r \in [\xi, 1], \tag{22}$$

where $w(z)$ is an analytic function with non-vanishing derivative in $\mathcal{B}$. The outer boundary can be defined parametrically by $u = \mathrm{Re}[w(e^{i\phi})], v = \mathrm{Im}[w(e^{i\phi})]$. We aim to solve the following stationary Dirac equation:

$$-i\hat{\boldsymbol{\sigma}} \cdot \boldsymbol{D}_{uv}\Psi(u, v) = k\Psi(u, v), \tag{23}$$

together with the boundary condition

$$\Psi_2/\Psi_1|_{\partial\mathcal{B}} = \mathrm{sgn}(V)ie^{i\theta},$$

where $k \equiv E/\hbar v$, $\Psi = [\Psi_1, \Psi_2]^{\mathrm{T}}$ denotes the spinor wave-function, $\boldsymbol{D}_{uv} = \boldsymbol{\nabla}_{uv} + \boldsymbol{a}(u, v)$, and $\boldsymbol{a} = 2\pi\boldsymbol{A}/\Phi_0$ denotes the normalized magnetic vector potential, with

$$\boldsymbol{a}(u, v) = \alpha(\partial g/\partial v, -\partial g/\partial u, 0)$$

being the contours of the scalar function $g(u, v)$ given by $\Delta_{uv}g = -2\pi\delta(u, v)$. Under the operator $-i\hat{\boldsymbol{\sigma}} \cdot \boldsymbol{D}_{uv}$, Eq. (23) becomes

$$\left[-\boldsymbol{D}_{uv}^2\mathbf{1} + 2\pi\alpha\delta(u, v)\hat{\sigma}_z\right]\Psi(u, v) = k^2\Psi(u, v), \tag{24}$$

where

$$\boldsymbol{D}_{uv}^2 = \Delta_{uv} + 2i\alpha\left(\frac{\partial g}{\partial v}\partial_u - \frac{\partial g}{\partial u}\partial_v\right) \qquad - \alpha^2\left[\left(\frac{\partial g}{\partial u}\right)^2 + \left(\frac{\partial g}{\partial v}\right)^2\right]. \tag{25}$$

Using the conformal mapping

$$\begin{pmatrix} \Delta \\ \frac{\partial F}{\partial y}\partial_x - \frac{\partial F}{\partial x}\partial_y \\ \left(\frac{\partial F}{\partial x}\right)^2 + \left(\frac{\partial F}{\partial y}\right)^2 \\ \delta(\boldsymbol{r}) \end{pmatrix} = \left|\frac{\mathrm{d}w}{\mathrm{d}z}\right|^2 \begin{pmatrix} \Delta_{uv} \\ \frac{\partial g}{\partial v}\partial_u - \frac{\partial g}{\partial u}\partial_v \\ \left(\frac{\partial g}{\partial u}\right)^2 + \left(\frac{\partial g}{\partial v}\right)^2 \\ \delta(u,v) \end{pmatrix}$$

to transform Eq. (24) to the circular ring domain in the z-plane, together with the definitions $F(\boldsymbol{r}) \equiv f(u,v)$ and $\Psi'(\boldsymbol{r}) = \Psi(u,v)$, we obtain the following form of Eq. (23):

$$\left[\boldsymbol{D}^2\mathbf{1} - 2\pi\alpha\delta(\boldsymbol{r})\hat{\sigma}_z\right]\Psi'(\boldsymbol{r}) + k^2\left|\frac{\mathrm{d}w}{\mathrm{d}z}\right|\Psi'(\boldsymbol{r}) = 0. \tag{26}$$

The ring topology guarantees that any singular solutions that are divergent at the origin [6–8] arising from the singular Zeeman-like term introduced in Eqs. (24) and (26) can be avoided.

To solve Eq. (26), we expand Ψ' in terms of the eigenfunctions $\{\psi_j(r,\theta), j = 1, \cdots\}$ of the circular ring domain in the presence of a central flux line:

$$\Psi'(r,\theta) = \sum_{j=1}^{\infty} c_j\psi_j(r,\theta), \tag{27}$$

where c_j's are the expansion coefficients. Substituting Eq. (27) into Eq. (26), together with the orthogonality of ψ_j, we obtain

$$\frac{\nu_i}{k^2} - \sum_j M_{ij}\nu_j = 0, \tag{28}$$

where $\nu_i = \mu_i c_i$,

$$M_{ij} = \frac{1}{\mu_i}\langle i|T(r,\theta)|j\rangle\frac{1}{\mu_j}, \tag{29}$$

and $T(r,\theta) = |\mathrm{d}w/\mathrm{d}z|^2$. Once the eigenvalues λ_n and eigenvectors $\boldsymbol{\nu}$ of the matrix (M_{ij}) are obtained, we get the complete solutions of Eq. (23) through the relations $k_n = 1/\sqrt{\lambda_n}$ and $c_i = \nu_i/\mu_i$. In actual computations, a truncated basis $\{\psi_j(r,\phi), j_{\min} \le j \le j_{\max}\}$ can be used, where $j_{\min}$ denotes the first eigenstate. The conformal mapping based method can yield a large number of energy levels and the corresponding eigenstates with high accuracy [5].

B. Energy level statistics

To demonstrate the working of our conformal-mapping based method to calculate the eigenenergies and eigenstates of the Dirac equation subject to AB flux, we set $\xi = 0.5$ for the circular ring domain $|z| \in [\xi, 1]$. We choose the following complex function $w(z)$ as a polynomial conformal map for the confinement domain:

$$w(z) = h[z + 0.05az^2 + 0.18a\exp(i\omega)z^5], \tag{30}$$

where $\omega \in [0, 2\pi)$ is a constant (we choose, somewhat arbitrarily, $\omega = \pi/2$), and $a \in [0,1]$ is the deformation parameter, which is also a control parameter to generate different types of classical

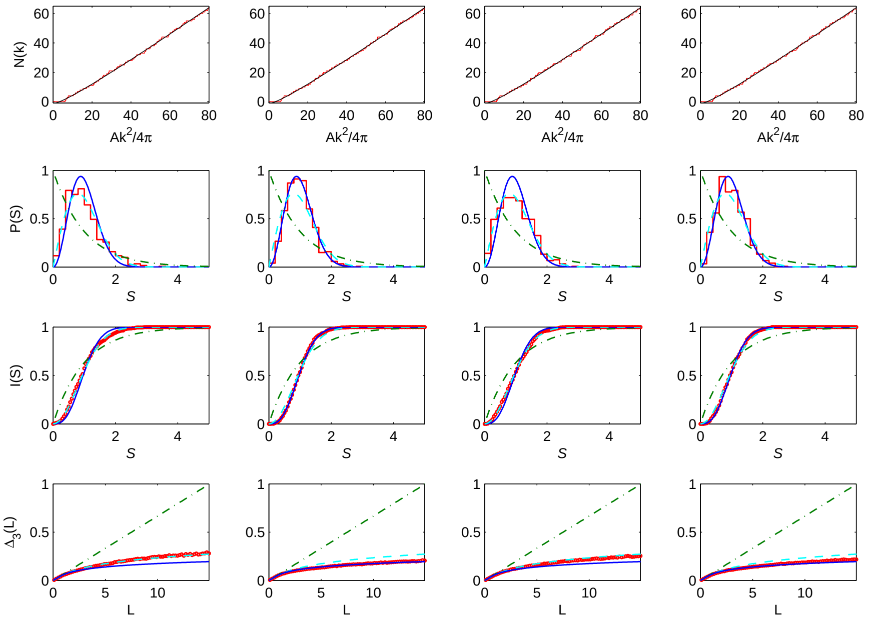

FIG. 2. Spectral statistics for the nonrelativistic AB chaotic billiard, where panels in columns 1-4 show, for quantum flux parameter $\alpha = 0, 1/4, 1/2, (\sqrt{5}-1)/2$, respectively, the unfolded level-spacing distribution $P(S)$, the cumulative level-spacing distribution $I(S)$, and the spectral rigidity Δ_3. In all panels, numerical data are represented by red line (or circle) and theoretical distribution curves for Poisson, GOE, and GUE statistics are plotted using green dash-dotted, cyan dashed, and blue solid curves, respectively.

dynamics. In particular, when a is increased from zero to unity, the deformed ring system will undergo a transition from being regular, mixed to fully chaotic. The normalization coefficient

$$h = \frac{1}{\sqrt{1 + \frac{a^2}{200}(1+\xi^2) + \frac{81a^2}{500}(1+\xi^2+\xi^4+\xi^6+\xi^8)}}$$

guarantees that the domain area is invariant for arbitrary values of the deformation parameters $\{a, \omega, \xi\}$. Justifications for choosing this particular transformation to generate ring domains with distinct types of classical dynamics are as follows. Firstly, to our knowledge, the form of $w(z)$ presented in Eq. (30) is the simplest conformal map that can result in classically chaotic billiards with the topology of a ring but without any geometric symmetries. For example, choosing $\{a = 1, \omega = \pi/2, \xi = 0.5\}$ fulfills this requirement [9]. Secondly, the conformal map also has the advantage of being analytically amenable. Thirdly, since the matrices (M_{ij}) are nearly diagonal, the associated computational cost is minimal. Fourthly, as classical dynamics is concerned, this family of deformed rings is especially appealing as transitions from regular to mixed and finally to fully chaotic motions can be realized by tuning a single parameter, a, from 0 to 1.

To validate our method, we calculate the level-spacing statistics, which are believed to exhibit universal characteristics for quantum systems, nonrelativistic [10, 11] or relativistic [12], that exhibit chaos in the classical limit. In particular, let $\{k_n | n = 0, 1, 2, \cdots\}$ denote the non-

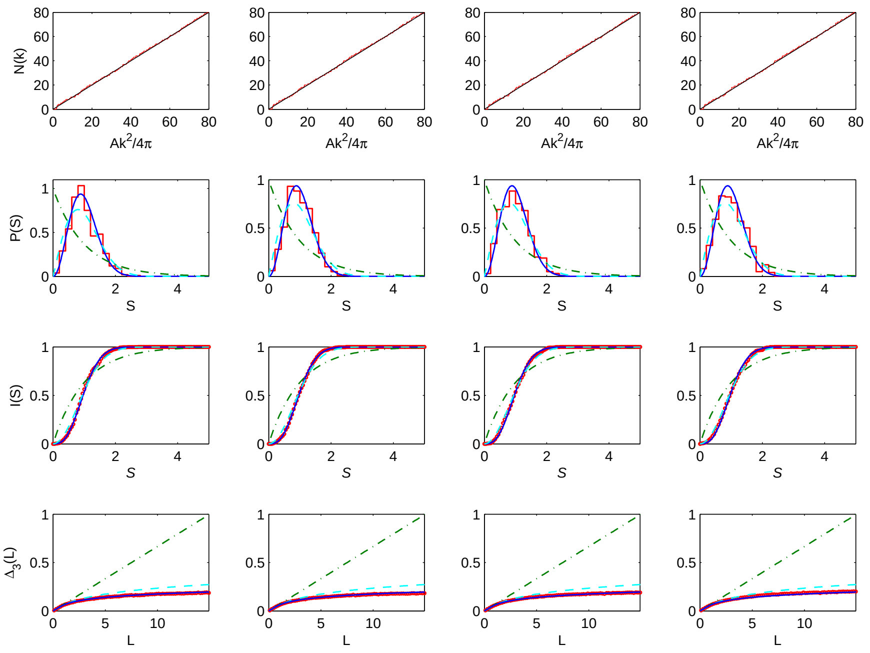

FIG. 3. Spectral statistics for the *relativistic* AB chaotic billiard. The legends are the same as in Fig. 2.

decreasing positive wave-number sequence of a quantum billiard system. According to the Weyl formula [10, 11] that has the same form regardless of presence or absence of a magnetic flux, the smoothed wave-vector staircase function is given by

$$\langle \mathcal{N}(k) \rangle = \frac{\mathcal{A}k^2}{4\pi} + \gamma \frac{\mathcal{L}k}{4\pi} + \cdots , \tag{31}$$

where $\mathcal{A}$ and $\mathcal{L}$ are the area and the perimeter of the billiard, respectively, $\gamma = -1$ (or 1) holds for Dirichlet (or Neumann) boundary conditions, and $\gamma = 0$ holds for massless Dirac fermion billiards [1]. Define $x_n \equiv \langle \mathcal{N}(k_n) \rangle$ as the unfolded spectra scaled in units of the mean-level spacing. Let $S_n = x_{n+1} - x_n$ be the nearest-neighbor spacing and $P(S)$ be the probability distribution of S [i.e., $P(S)dS$ is the probability that a spacing S lies between S and $S + dS$]. It has been known that, for classically integrable systems, the level spacing distribution is Poisson: $P(S) \sim \exp(-S)$. For classically chaotic systems that possess time-reversal symmetry but no geometrical symmetry, the level-spacing distributions follow the GOE (Gaussian orthogonal ensemble) statistics: $P(S) = (\pi/2)S \exp(-\pi S^2/4)$. For chaotic systems without time-reversal symmetry, $P(S)$ obeys the GUE (Gaussian unitary ensemble) statistics: $P(S) = (32/\pi)S^2 \exp(-4S^2/\pi)$. Given $P(S)$, the corresponding cumulative level-spacing distribution can be obtained from $I(S) = \int_0^S \mathrm{d}S' P(S')$. Different types of level-spacing statistics can also be distinguished by using the Δ_3 statistics [10, 11].

To give an example, we choose the parameters $a = 1, \omega = \pi/2, \xi = 0.5$. Diagonalizing the matrix (M_{ij}) gives about 2000 levels for each of the four values of the magnetic flux $\alpha = 0, 1/4, 1/2, (\sqrt{5} - 1)/2$. We use the lowest 600 levels to calculate the spectral statistics. Figures 2 and 3 show the statistics from the non-relativistic (Schrödinger) and relativistic (Dirac) AB chaotic billiards, respectively. As predicted, we observe GUE statistics for the relativistic quantum

billiard because of the persistent T-breaking by the confinement (Fig. 3). Deviations from the GUE statistics are observed for the zero-flux case (the 1st column) and the "false T-breaking" case with $\alpha = 1/2$ (the 3th column) for nonrelativistic quantum billiard (Fig. 2).

SUPPLEMENTARY NOTE 3: EQUALITY OF INTERFACT CURRENT DENSITY FROM EQS. (7) AND (8) IN THE MAIN TEXT

The analyzable model in the main text is a metal-insulator step junction (which is relevant to the closed rings case), where the insulator region is denoted as II and the metal region as I. Physically, the Hall-like current at the interface can be attributed to the T-breaking mass potential in the insulator region.

At $x = 0$, Eqs. (7) and (8) are identical, which can be seen, as follows. In the main text, we have

$$\tan\gamma = \frac{\lambda_1 - \lambda_2 \sin\theta_0}{\lambda_2 \cos\theta_0} = \frac{1 - \lambda\sin\theta_0}{\lambda\cos\theta_0}. \quad (32)$$

Setting $x = 0$, we have

$$\begin{aligned}(J_D^I)_y &= v[2\sin\theta_0 + 2\sin(2\gamma + \theta_0)],\\ (J_D^{II})_y &= v\frac{4\cos^2\gamma}{\lambda} \equiv v\frac{4\cos^2\gamma\cos\theta_0}{\lambda\cos\theta_0}.\end{aligned}$$

From Eq. (32), we have

$$\frac{1}{\lambda\cos\theta_0} = \tan\gamma + \tan\theta_0.$$

Substituting this into the expression for $(J_D^{II})_y$, we obtain

$$\begin{aligned}(J_D^{II})_y &= 4v(\tan\gamma + \tan\theta_0)\cos^2\gamma\cos\theta_0\\ &= 4v(\sin\gamma\cos\theta_0 + \cos\gamma\sin\theta_0)\cos\gamma\\ &= v[2\sin(2\gamma + \theta_0) + 2\sin\theta_0]\\ &= (J_D^I)_y.\end{aligned}$$

[1] M. V. Berry and R. J. Mondragon, Proc. Roy. Soc. Lond. A **412**, 53 (1987).

[2] P. Recher, B. Trauzettel, A. Rycerz, Y. M. Blanter, C. W. J. Beenakker, and A. F. Morpurgo, Phys. Rev. B **76**, 235404 (2007).

[3] M. Robnik, J. Phys. A **17**, 1049 (1984).

[4] M. V. Berry and M. Robnik, J. Phys. A **19**, 649 (1986).

[5] H. Xu, L. Huang, Y.-C. Lai, and C. Grebogi, Phys. Rev. Lett. **110**, 064102 (2013).

[6] M. G. Alford and F. Wilczek, Phys. Rev. Lett. **62**, 1071 (1989).

[7] P. d. S. Gerbert, Phys. Rev. D **40**, 1346 (1989).

[8] C. R. Hagen, Phys. Rev. Lett. **64**, 503 (1990).

[9] C. Stelzer and W. Schweizer, Phys. Rev. B **62**, 7275 (2000).
[10] H. J. Stöckmann, *Quantum chaos: an introduction* (Cambridge University Press, Cambridge, England, 1999).
[11] F. Haake, *Quantum Signatures of Chaos, 2nd Edition* (Springer, Berlin, 2001).
[12] L. Huang, Y.-C. Lai, and C. Grebogi, Phys. Rev. E **81**, 055203 (2010).
